# A network meta-analysis of *probiotics* in the treatment of childhood asthma

**DOI:** 10.3389/fped.2025.1637284

**Published:** 2025-09-25

**Authors:** Jiajia Sun, Meiyi Zhu, Ying Liu, Di Zhang

**Affiliations:** ^1^ Jiangxi University of Chinese Medicine, Nanchang, Jiangxi, China; ^2^ Department of Pediatrics, The Affiliated Hospital of Jiangxi University of Chinese Medicine, Nanchang, Jiangxi, China

**Keywords:** *probiotics*, conventional treatment, network meta-analysis, childhood, asthma

## Abstract

**Objective:**

This network meta-analysis aims to explore the efficacy and safety of *probiotics* in children with asthma and attempts to determine which *probiotics* are most effective in improving outcomes in children with asthma by ranking methods.

**Methods:**

A systematic search of Chinese and English databases, including China National Knowledge Infrastructure, Wanfang, VIP, PubMed, and Web of Science, was conducted from the establishment of the databases until July 2024 to screen for randomized controlled trials (RCTs) of *probiotics* in the treatment of childhood asthma. Lung function was used as the primary outcome measure, and secondary outcome measures included the total clinical response rate, recurrence rate, immune factors, cytokines, and Childhood Asthma Control Test (C-ACT) score. Data processing and analysis were performed using RevMan 5.4 and Stata 17.0 software.

**Results:**

A total of 34 RCTs were included, involving 3,839 participants and 13 interventions. Our analysis showed that conventional treatment combined with *probiotics* improved outcome indicators in children with asthma better than conventional treatment. Conventional treatment combined with *Bifidobacterium*–*Lactobacillus* triplex live bacteria had the highest probability of being the optimal intervention in terms of increasing FEV_1_% and recurrence rate. Conventional treatment combined with *Bifidobacterium adolescentis* had the highest probability of being the optimal intervention in increasing FEV_1_. Conventional treatment combined with *Lactobacillus* tablets had the highest probability of being the optimal intervention in increasing peak expiratory flow. Conventional treatment combined with *Bacillus subtilis* diplex live bacteria had the highest probability of being the optimal intervention in improving the total clinical response rate. Conventional treatment combined with *Bifidobacterium* quadruplex live bacteria had the highest probability of being the optimal intervention in reducing IL-4 and IL-33. Conventional treatment combined with *Bifidobacterium* triplex live bacteria had the highest probability of being the optimal intervention in improving the C-ACT score.

**Conclusion:**

*Probiotics* are effective in treating childhood asthma, and the therapeutic effect of conventional treatment combined with *probiotics* is superior to that of conventional treatment alone. Therefore, *probiotics* can be selected as appropriate in the clinical treatment of childhood asthma. However, the overall quality of the evidence was at most low or moderate, suggesting that the certainty of the evidence for *probiotics* in treating childhood asthma was low.

**Systematic Review Registration:**

https://www.crd.york.ac.uk/PROSPERO/, PROSPERO CRD42024617940.

## 1 Background

Asthma is a heterogeneous disease characterized by chronic airway inflammation and airway hyperresponsiveness. Clinically, it is characterized by recurrent episodes of wheezing, coughing, shortness of breath, and chest tightness, which often occur or worsen at night and/or in the early hours of the morning. The cumulative incidence rates of childhood asthma in 1990, 2000, and 2010 were 1.09%, 1.97%, and 3.02% (1) based on epidemiological investigations. While asthma hospitalizations and deaths have declined in some countries, asthma, especially childhood asthma, still imposes an unacceptable burden (2). Beta-agonists and leukotriene antagonists are commonly used in clinical practice to treat childhood asthma, but they are less effective in regulating immune function, so childhood asthma is prone to relapse (3) and eventually develops into adult asthma (4).

With further research on the gut microbiota, researchers have found that *probiotics* can improve lung function in children with asthma, increase the total clinical response rate, improve quality of life, and reduce the recurrence rate (5) of asthma. The types of *probiotics* involved in these reports on treating childhood asthma are diverse. Currently, there is a lack of comparative studies on the efficacy of different interventions, and there is no mention of probiotic types, specific usage, or treatment regimens (6) in the relevant guideline for childhood asthma. As a result, clinicians often rely on subjective experience when making treatment decisions. Therefore, this study uses a network meta-analysis method to compare the effects of different probiotic interventions on children’s lung function, clinical efficacy, and reduction of recurrence rate, to provide evidence-based medical support for optimizing drug selection in clinical practice.

## 2 Data and analysis

### 2.1 Inclusion and exclusion criteria

The inclusion and exclusion criteria for this study were based on the PICOS strategy. Only randomized controlled trials (RCTs) reported in English or Chinese were included, with no geographical restrictions. Non-RCTs, animal experiments, case studies, expert opinions, and other types of studies were excluded.

This study included children with asthma aged 5–18 years. The age range of 5–18 years was selected to ensure diagnostic accuracy, as lung function testing is challenging in younger children, and to maintain homogeneity across studies. Intervention measures in the experimental group included *probiotics* (*Bifidobacterium* triplex live bacteria, *Bifidobacterium* quadruplex live bacteria, *Bifidobacterium*–*Lactobacillus* triplex live bacteria, *Bifidobacterium adolescentis*, *Saccharomyces boulardii*, *Bacillus subtilis diplex* live bacteria, *Lactobacillus* tablets, LF(LactobacillusBCRC 910259), LP(LactobacillusGMNL-133)) combined with conventional treatment. The control group received conventional treatment [standard treatment for childhood asthma based on contemporary guidelines, mainly including but not limited to: inhaled glucocorticoids (ICS), leukotriene receptor antagonists, short-acting/long-acting beta-2 agonists (SABA/LABA), anticholinergic drugs] or conventional treatment combined with placebo.

The exclusion criteria included (1) in cases of duplicate publications, only the earliest article (by publication date) is included, and if the same manuscript has been submitted to multiple publications, only the version with the most complete data is selected; (2) non-RCT and RCT studies not involving children; (3) reviews, discussions, empirical cases, animal experiments, etc.; (4) literature with missing data or where full text cannot be obtained; and (5) literature involving participants with coexisting organic diseases or other diseases.

### 2.2 Database and retrieval strategy

The system searched Chinese and English databases, including China National Knowledge Infrastructure, Wanfang, VIP, PubMed, and Web of Science. The search period was from the establishment of the database to 18 July 2024. Search terms include “Asthma,” “Cough-Variant Asthma,” “bronchial asthma,” “child*,” “Adolescence,” “*probiotics*,” “*Bifidobacterium*,” “Yeast fungus,” and “*Bacillus subtilis* double viable bacteria.”

### 2.3 Data screening and quality assessment

Two researchers (JS, MZ) independently conducted the literature search and screening using Zotero according to the established relevant criteria. They cross-checked the results, and any discrepancies were discussed with a third researcher until a consensus was reached.

Two researchers (DZ, YL) used the modified Jadad scale (7) and the Cochrane risk of bias tool (8) to assess the risk of deviation and quality of the included RCTs, and any disagreement was resolved by discussing with a third researcher.

### 2.4 Outcome measures

The primary outcome measures were lung function, including the ratio of forced expiratory volume in one second to forced vital capacity (FEV_1_/FVC), the percentage of forced expiratory volume in 1 s to the projected value (FEV_1_%), forced expiratory volume in 1 s (FEV_1_), and peak expiratory flow (PEF). The secondary outcome measures were (1) clinical total effective rate, (2) recurrence rate, (3) adverse reactions, (4) immune factors (IgA and IgE), (5) cytokines (IL-4 and IL-33), and (6) Childhood Asthma Control Test (C-ACT) score.

### 2.5 Data analysis

The network structure was star-shaped, and there were no direct comparisons, so this study relied on the transitivity hypothesis. We assessed transitivity by examining the following: (1) patient characteristics, all studies included children aged 5–18 years with mild-to-moderate persistent asthma; (2) conventional treatment principles, while specific drug regimens varied (ICS monotherapy, ICS + LABA, montelukast), all followed stepwise management principles per contemporaneous guidelines; and (3) study methodology, all were RCTs with similar outcome assessment methods.

#### 2.5.1 Outcome measures

For binary variables, relative risk (RR) was used as the effect size, whereas for continuous variables, standardized mean difference (SMD) was used as the effect size. All SMDs were based on endpoint values after intervention, and the effect size was expressed as a 95% confidence interval (95% CI).

#### 2.5.2 Analysis method

A network plot was plotted using Stata 17.0. All RCTs including one multi-arm trial (9) were included and analyzed as single units using multivariate meta-analysis models without manual splitting. For effect size calculation, Hedges’ *g* and its standard error (SE) were used for SMD. The comparative relationship between studies is presented visually through a network diagram. Subgroup analysis and meta-regression were conducted at high heterogeneity (*I*^2^ > 50%). The therapeutic effects of the intervention were ranked by the surface under the cumulative ranking curve (SUCRA), and a SUCRA difference of >15% with non-overlapping CI was considered a significant difference. Funnel plots were plotted to assess the small sample effect. The adequacy of the sample size was evaluated through assessment of the precision of effect estimates and confidence interval widths.

## 3 Results

### 3.1 Literature search results

**Figure 1:**
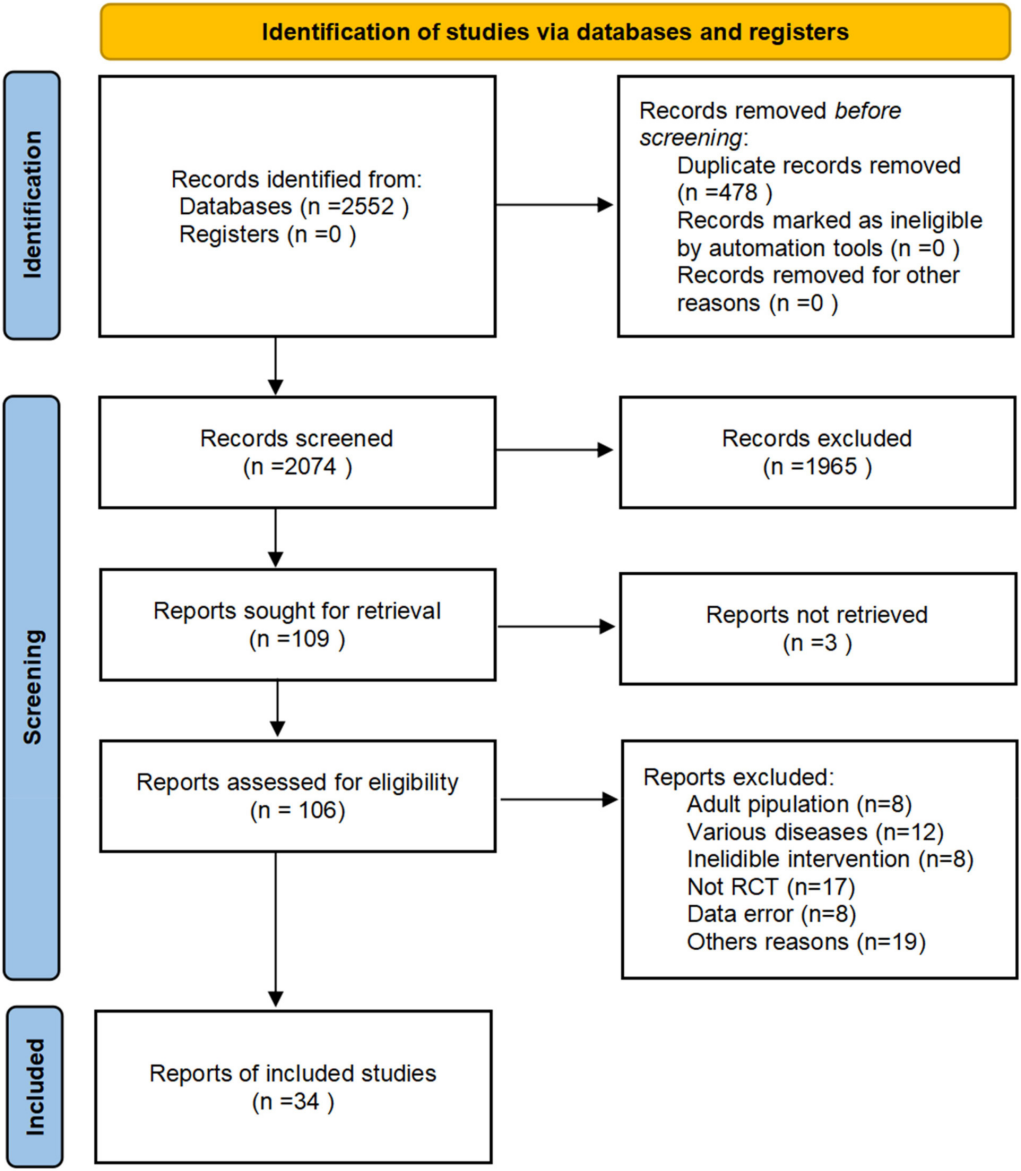
Literature inclusion process.

A total of 2,552 studies were retrieved from the database, and 478 duplicates were excluded. After screening for titles and abstracts, 109 records met the criteria for a full-text analysis. Among them, 72 studies were excluded by reading the full text. In total, 34 studies were included in this study (the literature screening process is shown in Figure 1).

### 3.2 Basic information and quality assessment of the included literature

**Figure 2:**
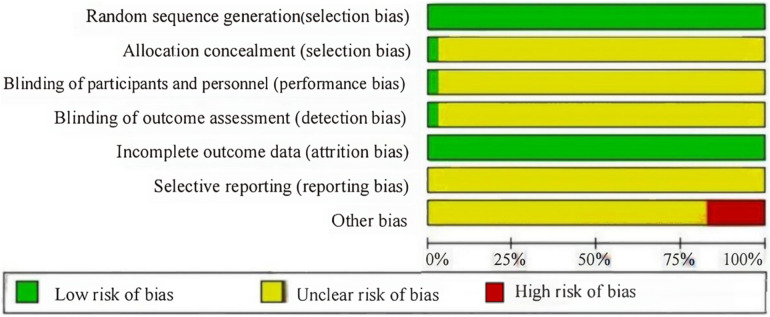
Cochrane risk of bias table.

A total of 34 studies were included, 33 from a Chinese database and 1 from an English database. This study involved 3,839 participants and 13 interventions. The basic information of the included studies and the evaluation of literature quality (Jadad score) are presented in Table 1. The Cochrane risk of bias tool was used to evaluate the risk of deviation in the included studies (Figure 2). All the included studies were RCTs. Only one study discussed the use of allocation concealment and blinding, two studies discussed data dropout, the rest did not report data dropout or loss to follow-up, and six studies had small sample sizes and were at high risk of bias.

**Table 1:** Basic characteristics of included literature as in original.

Inclusion of studies	Sample size (T/C)	Sex ratio (M/F)		Age (year)		Intervention		Treatments	Conclusion norm	Follow-up timing	Jadad rating
		T	C	T	C	T	C				
Sheng Ren 2020	42/42	25/17	24/18	9.15 ± 2.32	8.78 ± 1.74	1	12	4 weeks	EJH	6 months	2
Qibo Ma 2023	53/52	28/25	29/23	8.53 ± 1.77	8.47 ± 1.56	1	12	4 weeks	J	6 months	2
Rentao Wang 2012	78/78	No	No	No	No	1	12	12 weeks	EF	No	1
Dan Wang 2023	250/250	132/118	137/113	10.4 ± 2.9	10.9 ± 3.1	1	12	4 weeks	EJ	No	4
Chunhui Zang 2024	30/30	13/17	16/14	8.97 ± 1.00	9.10 ± 1.32	1	12	March	BEH	No	2
Yinfang Li 2023	31/31	16/15	15/16	9.9 ± 1.4	9.8 ± 1.6	1	12	6 months	ACDEHI	No	1
Qiaoxiang Xia 2022	46/46	24/22	21/25	6.84 ± 2.71	6.53 ± 2.59	1	12	4 weeks	CDE	No	2
Jia Li 2024	55/55	29/26	30/25	5.66 ± 1.25	5.62 ± 1.24	1	12	3 months	ABDEH	No	1
Jingfang Ma 2022	80/80	38/42	41/39	8.7 ± 1.2	8.8 ± 1.1	1	12	6 days	EJG	No	1
Bei Teng 2024	40/43	21/19	20/23	7.75 ± 1.82	7.75 ± 1.69	2	12	3 months	BH	3 months	2
Rongrong Tuo 2023	43/43	26/17	24/19	8.63 ± 1.52	8.22 ± 1.47	2	12	3 months	ACEJH	No	2
Shuxuan Chen 2021	55/55	24/31	21/34	7.71 ± 1.06	7.32 ± 1.21	2	12	1 months	I	No	2
Han Gao 2019	42/42	30/12	28/14	8.63 ± 1.73	8.70 ± 1.58	2	12	1 months	BCDGI	No	2
Chao Lu 2022	75/75	44/31	39/36	6.36 ± 4.54	6.89 ± 5.12	2	12	2 weeks	E	No	2
Deming Zhou 2020	50/50	25/25	24/26	9.24 ± 2.21	9.14 ± 2.22	2	12	No	BDFH	No	2
Lin Chen 2021	36/36	No	No	No	No	3	12	30 days	ACH	No	1
Shigang Li 2023	49/49	26/23	27/22	5.76 ± 1.42	5.46 ± 1.32	3	12	28 days	G	No	1
Qi Wang 2021	35/33	20/15	19/14	7.9 ± 1.9	7.9 ± 1.9	3	12	2 months	EI	3 months	3
Xue Li 2022	52/52	31/21	30/22	8.03 ± 1.26	7.85 ± 1.14	3	12	3 months	BCDEH	No	2
Shaoyan Wang 2012	30/30	No	No	No	No	3	12	3 months	EF	1 year	1
Yuwei Fu 2017	110/110	57/53	58/52	6.3 ± 0.6	6.1 ± 0.8	4	12	3 months	CDE	No	2
Yanfang Ma 2012	44/44	24/16	23/17	5.8 ± 1.1	5.6 ± 1.5	4	12	3 months	E	No	1
Linna Shen 2018	51/51	25/26	26/25	6.72 ± 2.38	6.38 ± 2.43	5	12	10 days	EG	No	1
Yan Yan Luo 2021	41/37	23/18	20/17	7.68 ± 3.91	7.70 ± 3.86	5	12	14 days	CE	No	2
Hongxia Dai 2023	55/55	32/13	30/25	5.90 ± 0.35	5.85 ± 0.30	5	12	14 days	EH	No	2
Weitu Chen 2020	54/54	33/21	29/25	Only the average age	Only the average age	6	12	6 weeks	ACDE	No	1
Bing Lv 2016	23/22	11/12	9/13	10.90 ± 2.10	10.10 ± 1.20	6	12	6 weeks	EGH	No	1
Wei Qin 2016	43/43	23/20	25/18	8.3 ± 3.3	9.8 ± 3.1	6	12	3 months	EF	half a year	1
Hui Li 2018	45/45	26/19	24/21	9.6 ± 3.2	8.8 ± 3.3	6	12	3 months	E	half a year	2
Yan Sun 2018	25/20	15/10	12/8	12.5 ± .2	12.4 ± 2.8	6	12	3 months	EF	No	1
Weiyu Xu 2023	52/52	30/22	31/21	7.18 ± 2.08	6.92 ± 1.79	7	12	4 weeks	EFG	June	3
Xiaofeng Yuan 2018	46/46	27/19	22/24	8.62 ± 1.73	8.48 ± 1.58	7	12	4 weeks	BDJH	No	2
Bingqi Zhang 2022	55/55	27/28	30/25	7.36 ± 2.16	7.86 ± 2.31	8	12	6 weeks	CDH	No	1
HuangChian-Feng 2018-1	38/35	22/16	18/17	7.68 ± 2.1	7.68 ± 2.50	9	13	3 months	GH	4 months	7
Huang Chian-Feng 2018-2	38/35	24/14	18/17	7.37 ± 2.34	7.68 ± 2.50	10	13	3 months	GH	4 months	7
Huang Chian-Feng 2018-3	36/35	19/17	18/17	7.00 ± 1.79	7.68 ± 2.50	11	13	3 months	GH	4 months	7

Intervention: (1) conventional treatment combined with *Bifidobacterium* triplex live bacteria; (2) conventional treatment combined with *Bifidobacterium* quadruplex live bacteria; (3) conventional treatment combined with *Bifidobacterium*–*Lactobacillus* triplex live bacteria; (4) conventional treatment combined with *Bifidobacterium adolescentis*; (5) conventional treatment combined with *Saccharomyces boulardii*; (6) conventional treatment combined with *probiotics*; (7) conventional treatment combined with *Bacillus subtilis* diplex live bacteria; (8) conventional treatment combined with *Lactobacillus* tablets; (9) conventional treatment combined with *LF (LactobacillusBCRC 910259)*; (10) conventional treatment combined with *LP (LactobacillusGMNL-133)*; (11) conventional treatment combined with *LP* *+* *LF*; (12) conventional treatment; (13) conventional treatment combined with *placebo*.

Outcome: (A) FEV_1_/FVC, (B) FEV_1_%, (C) FEV_1_, (D) PEF, (E) total clinical total effective rate, (F) recurrence rate, (G) immune factors, (H) cytokines, (I) C-ACT score, (J) adverse reaction.

### 3.3 Network meta-analysis

#### 3.3.1 Network diagrams of different interventions

**Figure 3:**
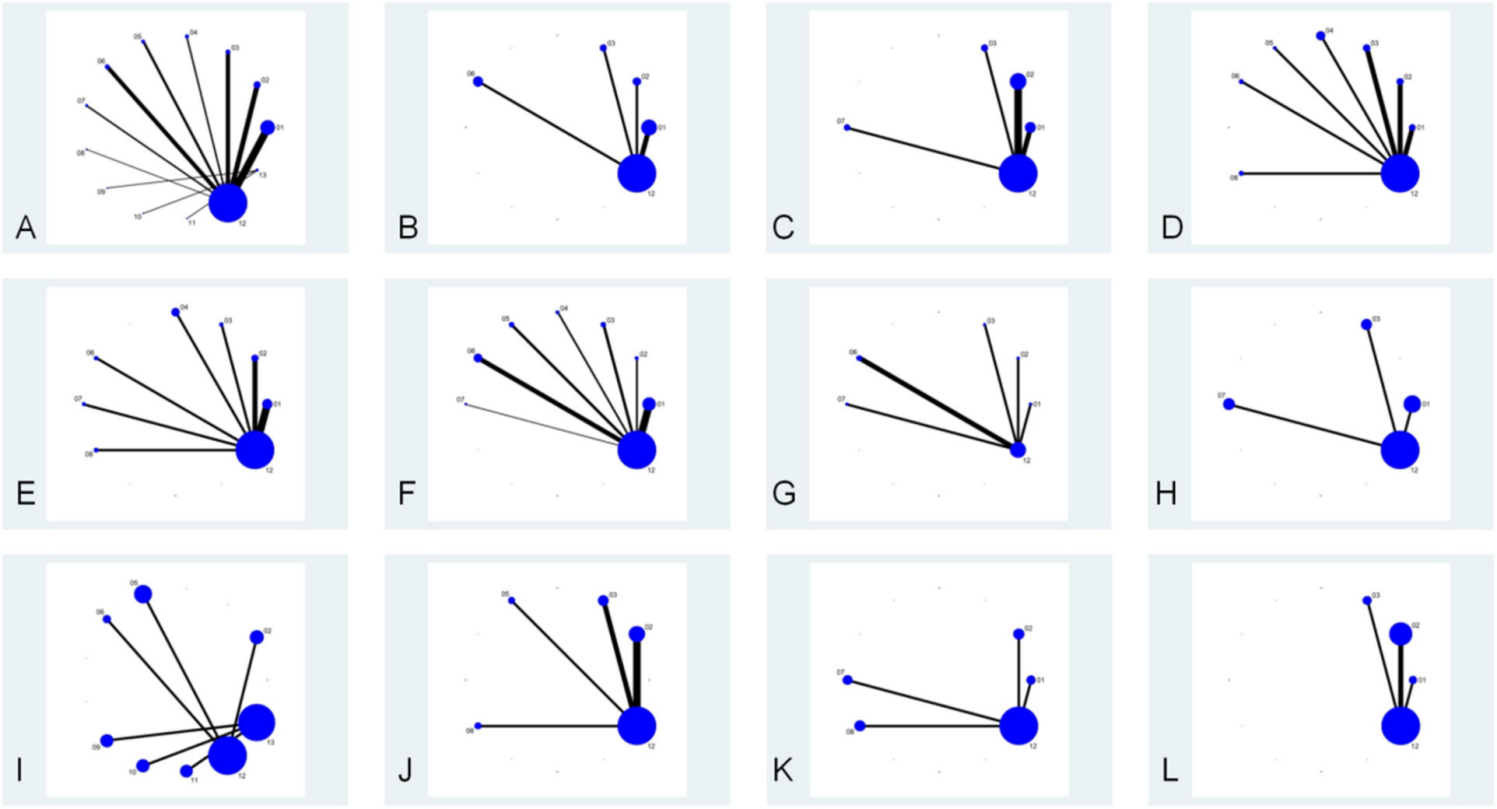
Network relationship diagram of each intervention, lung function, clinical effectiveness, recurrence rate, immune factors, cytokines, and C-ACT scores. The node “conventional treatment combined with probiotics” includes studies using probiotic supplements without specifying strain identity or commercial products. Each study was assigned to only one node based on intervention description clarity. **(A)** Each intervention; **(B)** FEV_1_/FVC; **(C)** FEV_1_%; **(D)** FEV_1_; **(E)** PEF; **(F)** total clinical effective rate; **(G)** recurrence rate; **(H)** IgA; **(I)** IgG; **(J)** IL-4; **(K)** IL-33; **(L)** C-ACT score. (1) Conventional treatment combined with *Bifidobacterium* triplex live bacteria; (2) conventional treatment combined with *Bifidobacterium* quadruplex live bacteria; (3) conventional treatment combined with *Bifidobacterium*–*Lactobacillus* triplex live bacteria; (4) conventional treatment combined with *Bifidobacterium adolescentis*; (5) conventional treatment combined with *Saccharomyces boulardii*; (6) conventional treatment combined with *probiotics*; (7) conventional treatment combined with *Bacillus subtilis* diplex live bacteria; (8) conventional treatment combined with *Lactobacillus* tablets; (9) conventional treatment combined with *LF (LactobacillusBCRC 910259)*; (10) conventional treatment combined with *LP (LactobacillusGMNL-133)*; (11) conventional treatment combined with *LP* *+* *LF*; (12) conventional treatment; (13) conventional treatment combined with *placebo*.

The network diagram of conventional treatment combined with *probiotics* vs. conventional treatment is shown in Figure 3. Dots represent intervention measures, and arms represent comparative studies. The larger the dots, the larger the sample size included. The thicker the arms, the higher the contribution of the corresponding comparison studies (related to the number of references and sample size). Among them, the combination of conventional treatment and *Bifidobacterium* triplet live bacteria has the most direct comparisons with conventional treatment.

#### 3.3.2 Inconsistency test

Due to the open network structure without closed loops, inconsistency testing was neither feasible nor necessary. We employed a consistency model throughout, assuming agreement between direct and indirect evidence.

### 3.4 Outcome measures

#### 3.4.1 Primary outcome measures

##### 3.4.1.1 FEV_1_/FVC

**Figure 4:**
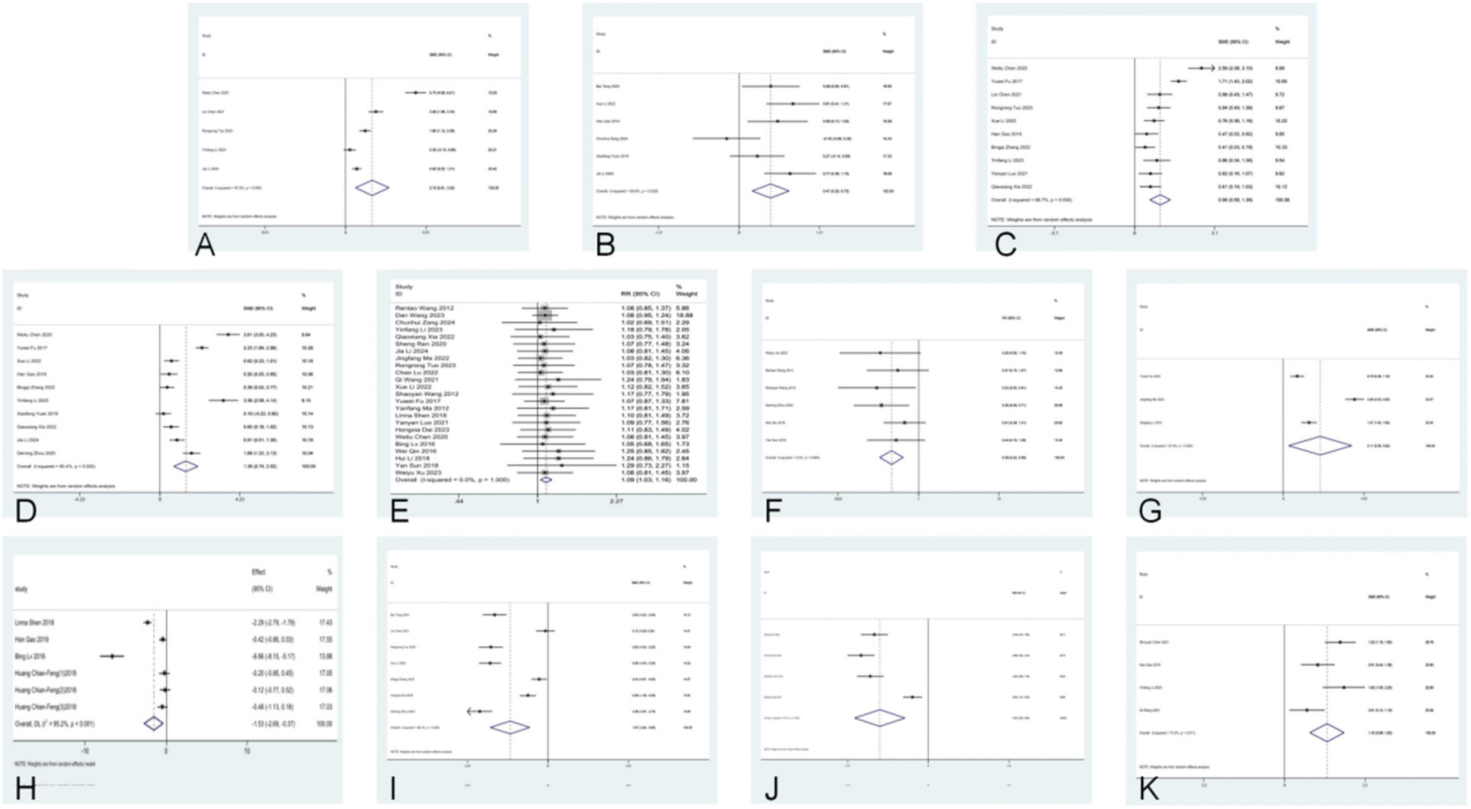
Forest plots for lung function, clinical effectiveness, recurrence rate, immune factors, cytokines, and C-ACT scores. **(A)** FEV_1_/FVC; **(B)** FEV_1_%; **(C)** FEV_1_; **(D)** PEF; **(E)** total clinical effective rate; **(F)** recurrence rate; **(G)** IgA; **(H)** IgG; **(I)** IL-4; **(J)** IL-33; **(K)** C-ACT score.

**Figure 5:**
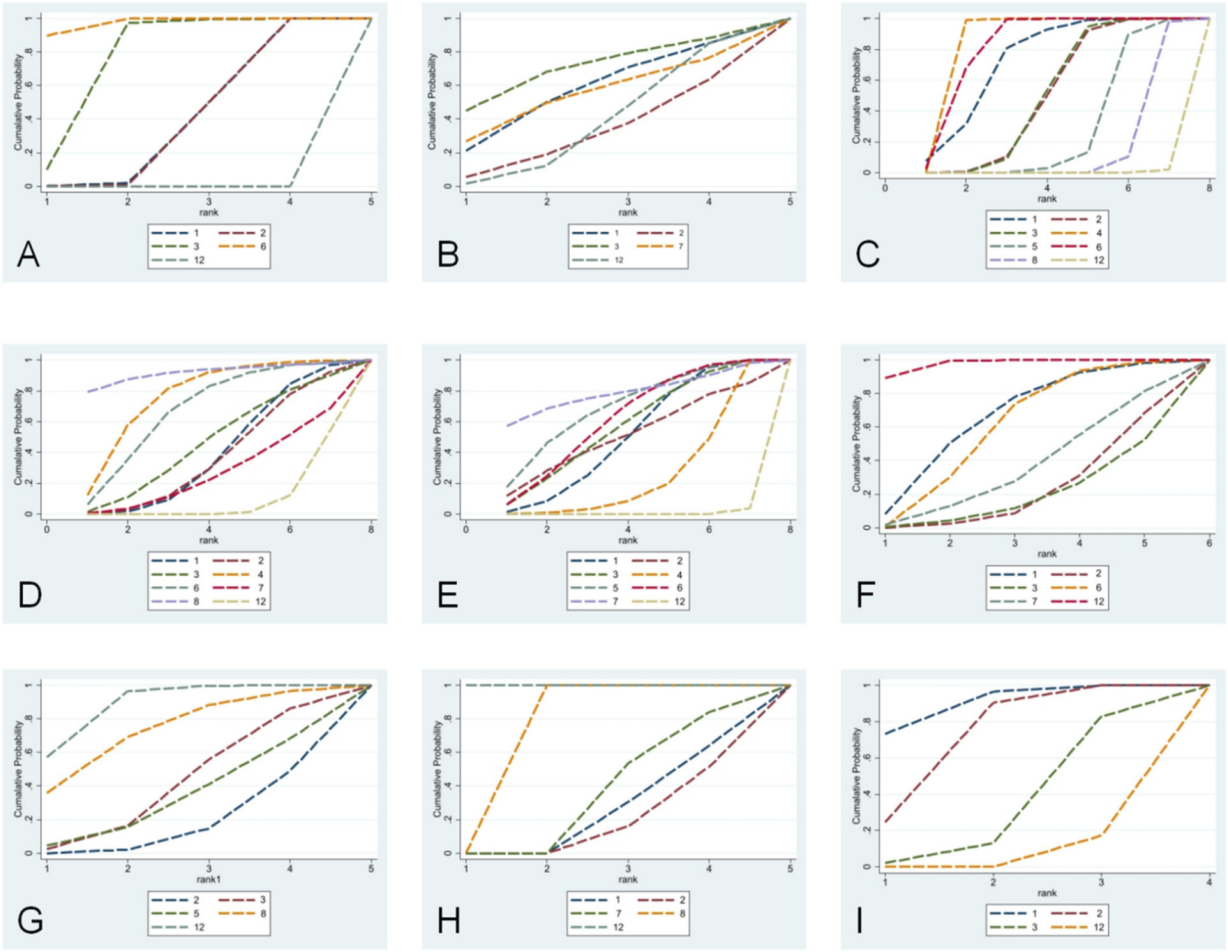
SUCRA plots for lung function, clinical effectiveness, recurrence rate, cytokines, and C-ACT scores. **(A)** FEV_1_/FVC; **(B)** FEV_1_%; **(C)** FEV_1_; **(D)** PEF; **(E)** total clinical effective rate; **(F)** recurrence rate; **(G)** IL-4; **(H)** IL-33; **(I)** C-ACT score. (1) Conventional treatment combined with *Bifidobacterium* triplex live bacteria; (2) conventional treatment combined with *Bifidobacterium* quadruplex live bacteria; (3) conventional treatment combined with *Bifidobacterium*–*Lactobacillus* triplex live bacteria; (4) conventional treatment combined with *Bifidobacterium adolescentis*; (5) conventional treatment combined with *Saccharomyces boulardii*; (6) conventional treatment combined with *probiotics*; (7) conventional treatment combined with *Bacillus subtilis* diplex live bacteria; (8) conventional treatment combined with *Lactobacillus* tablets; (9) conventional treatment combined with *LF (LactobacillusBCRC 910259)*; (10) conventional treatment combined with *LP (LactobacillusGMNL-133)*; (11) conventional treatment combined with *LP* *+* *LF*; (12) conventional treatment; (13) conventional treatment combined with *placebo*.

Five studies (10–14) were included in the analysis for the pooled estimation analysis of FEV_1_/FVC, and the network diagram (Figure 3) involved four interventions involving a total of 438 participants. Funnel plots were plotted, and Egger’s test was performed to assess publication bias for the metric. The funnel plots were visually asymmetrical, and the Egger’s test was performed with a *P*-value of 0.049, which is statistically significant but close to the critical value, suggesting the possibility of potential publication bias, considering the small sample effect and the higher possibility of publication bias in combination. The heterogeneity test for the forest plot (Figure 4) showed *I*^2^ = 97%, *P* = 0.000, suggesting high heterogeneity among the studies. Meta-regression and subgroup analyses were conducted on the included studies, and heterogeneity did not decrease. It is speculated that the sources of heterogeneity may be publication bias, small sample size, and low-quality literature. The random-effects model was used for statistical analysis. The combined SMD value in this study was 2.18, 95% CI (0.81, 3.55), and the difference was statistically significant, suggesting that *probiotics* are effective in enhancing FEV_1_/FVC function in children with asthma. In the SUCRA plot of the four interventions affecting FEV_1_/FVC (Figure 5), conventional treatment combined with *probiotics* has the largest area, indicating the highest probability of being the optimal intervention in improving the FEV_1_/FVC index in children with asthma.

##### 3.4.1.2 FEV_1_%

Six studies (11, 15–20) were included in the analysis for the pooled estimate analysis of FEV_1_%, and the network diagram (Figure 3) involved six interventions involving a total of 527 participants. Funnel plots were plotted, and Egger's test was performed to assess publication bias in the indicator. The funnel plots were significantly asymmetrical, suggesting a potential for publication bias. Egger's test was performed with a *P*-value of 0.058, which was not statistically significant, considering the marginal risk of publication bias in combination with the two. The heterogeneity test for the forest plot (Figure 4) showed *I*^2^ = 59.8%, *P* = 0.029, suggesting significant heterogeneity among the studies. Meta-regression and subgroup analysis of the included studies showed no decline in heterogeneity, suggesting that the sources of heterogeneity might be publication bias, small sample size, and low-quality literature. The random-effects model was used for statistical analysis, with a combined SMD value of 0.47, 95% CI (0.20, 0.75), and the difference was statistically significant, suggesting that *probiotics* are effective in improving FEV_1_% function in children with asthma. In the SUCRA ranking chart of the six interventions affecting FEV_1_% (Figure 5), conventional treatment combined with *Bifidobacterium Lactobacillus* triplex live bacteria has the largest area, indicating the highest probability of being the optimal intervention in improving the FEV_1_% index in children with asthma.

##### 3.4.1.3 FEV_1_

Ten studies (10, 12–14, 17, 19, 21–24) reported FEV_1_, and the network diagram (Figure 3) involved eight interventions involving a total of 1,010 subjects. To assess publication bias for the indicator, funnel plots were plotted, and Egger's test was conducted. The funnel plots were symmetrically distributed, and Egger's test was performed with a *P*-value of 0.757, which was not statistically significant. Considering the lack of significant publication bias and the small sample effect, the results showed a lower risk of selective publication. The heterogeneity test for the forest plot (Figure 4) showed *I*^2^ = 88.7%, *P* = 0.000, suggesting significant heterogeneity among the studies. Meta-regression and subgroup analysis were conducted on the included studies, and the conclusion was that the number of strains (i.e., single strain and compound strain) was one of the sources of heterogeneity. After excluding the single strain, the heterogeneity test yielded *I*^2^ = 0.00%, *P* = 0.610. Two studies (14, 22) were identified as sources of heterogeneity. After excluding these two studies, the heterogeneity test yielded *I*^2^ = 0.00%, *P* = 0.510. Therefore, it is presumed that the sources of heterogeneity are these two studies and the number of strains. Using the *random-effects model* for statistical analysis, the combined SMD value was 0.99, 95% CI (0.59, 1.39), and the difference was statistically significant, suggesting that *probiotics* are effective in enhancing FEV_1_ function in children with asthma. In the SUCRA ranking chart of the eight interventions affecting FEV_1_ (Figure 5), conventional treatment combined with *Bifidobacterium adolescentis* has the largest area, indicating the highest probability of being the optimal intervention in improving FEV_1_ indicators in children with asthma.

##### 3.4.1.4 PEF

Ten studies (10, 11, 14, 17–22, 24) reported PEF, and the network diagram (Figure 3) involved seven intervention measures, involving a total of 1,076 subjects. To assess publication bias for the indicator, funnel plots were plotted, and Egger's test was conducted. The funnel plots were relatively symmetrical in distribution, and Egger's test was performed with a *P*-value of 0.157, which was not statistically significant. No small sample effect was found. Considering the low risk of interference from publication bias, the results were relatively robust, and the reliability of the conclusion was guaranteed to some extent. The heterogeneity test for the forest plot (Figure 4) showed *I*^2^ = 96.8%, *P* = 0.000, suggesting significant heterogeneity among the studies. Meta-regression and subgroup analysis were conducted on the included studies, suggesting that the sources of heterogeneity might be publication bias, small sample sizes, and low-quality literature. Therefore, a random-effects model was used for statistical analysis. The combined SMD value was 1.76, 95% CI (0.74, 2.78), and the difference was statistically significant, suggesting that *probiotics* are effective in improving PEF function in children with asthma. In the SUCRA ranking chart of the seven interventions affecting PEF (Figure 4), conventional treatment combined with *Lactobacillus* tablets has the largest area, indicating the highest probability of being the optimal intervention in improving PEF indicators in children with asthma.

#### 3.4.2 Secondary outcome measures

##### 3.4.2.1 Clinical total effective rate

Twenty-four studies (5, 10–12, 14, 15, 19, 21–23, 25–38) reported the clinical total effective rate, and the network diagram (Figure 3) involved 11 interventions involving a total of 2,768 participants. To assess the publication bias of this indicator, funnel plots were plotted, and Egger's test was conducted. The funnel plots were asymmetrically distributed left and right, with a small portion close to the bottom of the funnel, suggesting a possible small sample effect and publication bias. Egger's test was performed with a *P*-value of 0.012, and the slope was statistically significant, considering the small sample effect and publication bias in combination with the two. The heterogeneity test for the forest plot (Figure 4) showed *I*^2^ = 0%, *P* = 1.000, suggesting that the heterogeneity among the studies was small. A fixed-effect model was used for statistical analysis. The combined RR value was 1.18, 95% CI (1.14, 1.21), and the difference was statistically significant, suggesting that *probiotics* were effective in the clinical total effective rate of treating childhood asthma. In the SUCRA ranking chart of 13 interventions affecting total clinical efficacy (Figure 5), conventional treatment combined with *Bacillus subtilis* diplex live bacteria has the largest area in the SUCRA chart, indicating the highest probability of being the optimal intervention in improving the total clinical efficacy index in children with asthma.

##### 3.4.2.2 Recurrence rate

Six studies (18, 25, 30, 35, 37, 38) reported recurrence rates, and the network diagram (Figure 3) involved six interventions involving a total of 544 subjects. To assess the publication bias of the indicator, funnel plots were plotted, and Egger's test was conducted. The funnel plots were asymmetrically distributed, mostly near the bottom of the funnel, suggesting a possible small sample effect. Egger's test was performed with a *P*-value of 0.069, which was not statistically significant, considering the marginal risk of the small sample effect in combination with the two. Subgroup analysis by follow-up duration (0–6 months vs. 6–12 months) showed that follow-up time was not a source of heterogeneity. The heterogeneity test for the forest plot (Figure 4) showed *I*^2^ = 0.00%, *P* = 0.528, suggesting that the heterogeneity among the studies was small. A fixed-effect model was used for statistical analysis. The combined RR value was 0.31, 95% CI (0.21, 0.48), and the difference was statistically significant, suggesting that *probiotics* are effective in reducing the recurrence rate of asthma in children. In the SUCRA ranking chart of the six interventions affecting the clinical total effective rate (Figure 5), conventional treatment combined with *Bifidobacterium*–*Lactobacillus*–*Enterococcus* triple probiotic has the smallest area, indicating the highest probability of being the optimal intervention in reducing the recurrence rate of asthma in children.

##### 3.4.2.3 Adverse reactions

Six studies (3, 5, 12, 20, 26, 27) reported adverse reaction rates, with no statistically significant difference. The combination of *probiotics* did not increase the risk of adverse events compared to conventional treatment alone. Adverse reactions are shown in Table 2 (*P* > 0.05).

**Table 2:** Adverse reactions.

Included studies	Experimental group		Control group	
	Adverse reactions	Total number	Adverse reactions	Total number
Sheng Ren 2020	3	42	5	42
Qibo Ma 2023	3	53	6	52
Jingfang Ma 2022	5	80	11	80
Rongrong Tuo 2023	7	43	6	43
Dan Wang 2023	16	250	19	250
Xiaofeng Yuan 2018	4	46	5	46
Total	38	514	52	513

##### 3.4.2.4 Immune factors IgA and IgE

Three studies (27, 38, 39) reported IgA, and the network diagram (Figure 3) involved four interventions involving a total of 362 subjects. The heterogeneity test for the forest plot (Figure 4) showed *I*^2^ = 97.9%, *P* = 0.000, suggesting significant heterogeneity among the studies. Due to the small sample size, meta-regression and subgroup analysis were not conducted, and the random-effects model was used for statistical analysis. The combined SMD value was 2.11, 95% CI (0.29, 3.92), and the difference was statistically significant. That is, conventional treatment combined with *probiotics* was better than conventional treatment in improving IgA.

Four studies (9, 17, 32, 34) reported IgE, and the network diagram (Figure 3) involved four interventions involving a total of 372 subjects. The heterogeneity test for the forest plot (Figure 4) showed *I*^2^ = 95.2%, *P* < 0.001, suggesting significant heterogeneity among the studies. Due to the small sample size, meta-regression and subgroup analysis were not conducted, and the random-effects model was used for statistical analysis. The combined SMD value was −1.53, 95% CI (−2.69, −0.37), and the difference was statistically significant. Conventional treatment combined with *probiotics* was better than conventional treatment in improving IgE.

##### 3.4.2.5 Cytokines IL-4, IL-33

Seven studies (12, 13, 16, 18, 19, 24, 33) reported IL-4. The network diagram (Figure 3) involved five intervention measures and a total of 665 subjects. Egger's test was performed on the data, with a *P*-value of 0.008, which was statistically significant, considering publication bias and small sample effects. The heterogeneity test for the forest plot (Figure 4) showed *I*^2^ = 95.4%, *P* = 0.000, suggesting significant heterogeneity among the studies. Meta-regression and subgroup analysis were conducted on the included studies, and the conclusion was that the Jadad score was one of the sources of heterogeneity. It was speculated that the sources of heterogeneity might be publication bias, small sample sizes, and low-quality literature. Random-effects models were used for statistical analysis. The combined SMD value was −1.87, 95% CI (−2.84, −0.9), and the difference was statistically significant, suggesting that conventional treatment combined with *probiotics* was better than conventional treatment in reducing IL-4. In the SUCRA ranking chart of the effects of IL-4 among the five interventions (Figure 5), conventional treatment combined with *Bifidobacterium* quadruplex live bacteria has the smallest area, indicating the highest probability of being the optimal intervention in improving IL-4 indicators in children with asthma.

Four studies (5, 18, 20, 24) reported IL-33, and the network diagram (Figure 3) involved five interventions involving a total of 386 subjects. Egger's test was performed with a *P*-value of 0.016, which was statistically significant, considering publication bias and small sample effect. The heterogeneity test for the forest plot (Figure 4) showed *I*^2^ = 95.4%, *P* = 0.000, suggesting significant heterogeneity among studies. Due to the small sample size, meta-regression and subgroup analysis were not conducted, and the random-effects model was used for statistical analysis. The combined SMD value was −1.94, 95% CI (−2.95, −0.93), and the difference was statistically significant. That is, conventional treatment combined with *probiotics* was better than conventional treatment in reducing IL-33. In the SUCRA ranking chart of the five interventions affecting IL-33 (Figure 5), conventional treatment combined with *Bifidobacterium* quadruplex live bacteria has the smallest area, indicating the highest probability of being the optimal intervention in improving IL-33 indicators in children with asthma.

##### 3.4.2.6 C-ACT score

Four studies (10, 17, 29, 40) reported C-ACT scores, with network diagrams (Figure 3) involving four interventions involving a total of 318 subjects. The heterogeneity test for the forest plot (Figure 4) showed *I*^2^ = 73.3%, *P* = 0.011, suggesting significant heterogeneity among the studies. Due to the small sample size, meta-regression and subgroup analysis were not conducted, and the random-effects model was used for statistical analysis. The combined SMD value was 1.16, 95% CI (0.69, 1.63), and the difference was statistically significant. That is, conventional treatment combined with *probiotics* significantly improved C-ACT scores compared to conventional treatment alone. In the SUCRA ranking chart of the four interventions affecting the C-ACT score (Figure 5), conventional treatment combined with *Bifidobacterium* triplex live bacteria has the largest area, indicating the highest probability of being the optimal intervention in improving the C-ACT index in children with asthma.

## 4 Discussion

The Global Burden of Disease Study 2016 estimates that more than 339 million people worldwide have asthma, and the age-standardized prevalence has increased by 3.6% since 2006. First asthma often appears in early childhood, with global prevalence rates of childhood wheezing and adolescent wheezing being 11.7% and 14.1% respectively, increasing by an average of 0.13% and 0.06% (41) annually. Professor David Strachan believes that too little exposure of children to environmental bacteria can lead to an increased (41) risk of allergies and asthma. Based on this hypothesis, *probiotics* have been proposed for the prevention and treatment (42) of allergic diseases such as asthma. This network meta-analysis focused on clinical outcomes rather than mechanistic pathways, as the included RCTs primarily reported clinical endpoints. While our analysis cannot establish strain-specific mechanisms from clinical outcome data alone, published mechanistic studies suggest different mechanisms for various strains. For example, *Bifidobacterium* can stimulate the Th1/Th2 balance and upregulate the secretion of IFN-γ, IL-4, and IL-12 in the spleen. *Lactobacillus plantarum* can reduce the number of innate immune cells in the lungs and the levels of IL-6 and TNF-α in bronchoalveolar lavage fluid and induce immunosuppressive Treg responses (43) in the lungs.

In clinical practice, it is necessary not only to follow the principles of relevant guidelines and diagnosis and treatment norms to improve the accuracy (44) of diagnosis of childhood asthma but also to seek safer and more effective treatment regimens. Although *probiotics* are often used to treat childhood asthma, there are currently no standard and effective probiotic usage regimens, and there is a lack of comparative studies on the efficacy of probiotic treatment regimens. How to select more effective probiotic usage regimens for different clinical conditions is a concern in clinical practice.

This study included only one English study that met the criteria [the single English study (9) showed consistent effect directions with Chinese studies, though formal statistical comparison was not possible], and the quality assessment showed an overall low risk of bias and a moderate risk of bias. The analysis suggests that *probiotics* combined with conventional treatment can significantly improve lung function in children with asthma, increase the clinical total effective rate, reduce the recurrence rate, and may be effective by increasing IgA (45), reducing IgE, IL-4 (46), and IL-33 levels. Although some studies (47) have reported that *probiotics* have no statistical significance in improving FEV_1_, PEF, and C-ACT scores, which may be related to the small number of studies included, the non-exclusion of comorbidities and young children, this study believes that the combination of *probiotics* is effective. At the same time, given the favorable safety profile demonstrated (no increase in adverse events) and efficacy shown, *probiotics* may be considered as adjunctive therapy in childhood asthma management.

This meta-analysis found issues of publication bias and small sample effect in indicators such as pulmonary function and total clinical effective rate (funnel plot and Egger's test). Publication bias may arise from the difficulty in publishing negative results and the preference for positive results, which undermines data symmetry. Small sample studies tend to overestimate the true effect, and the combination of these increases the risk of “false positives.” The field should vigorously promote large sample sizes, encourage the public release of negative results, and reduce bias throughout the entire process from research design to dissemination. Despite the bias limitations of this analysis, the core conclusions still provide a reference for clinical and scientific research, and more high-quality studies are needed in the future to verify them.

For highly heterogeneous outcome measures, we conducted subgroup analyses and meta-regression on intervention measures (i.e., strain types), strain quantities (single strains or complex strains), treatment courses, manufacturers, and Jadad scores, but did not analyze the dosage of *probiotics*. The reasons are as follows: (1) weak evidence base (dose range 1–650 × 10^6^ colony-forming units without established thresholds), making forced categorization prone to bias; (2) unclear dose–response relationships; (3) strain type and other factors having stronger confounding effects than dosage; and (4) studies focusing on strain–disease associations rather than dose optimization. The analysis did not identify the source of heterogeneity. It is presumed to be related to small sample size, publication bias, and low-quality literature. Therefore, given the high heterogeneity context, the pooled effect sizes provided should be considered exploratory estimates pending validation through high-quality RCTs. Due to the high risk of bias and heterogeneity, the quality of evidence suggesting *probiotics* improve FEV_1_/FVC, FEV_1_%, PEF, cytokines, and immune factors is low. The quality of evidence that *probiotics* improve FEV_1_, clinical total effective rate, and recurrence rate is moderate.

The validity of our indirect comparisons depends critically on the transitivity assumption. While we maintained consistency in age range (5–18 years) and disease severity (mild-to-moderate persistent asthma), variations in background conventional therapy may modify probiotic effects, representing a key limitation. (1) We strictly limited the subjects included in the study to children with asthma aged 5–18 years, ensuring comparability between studies in this dimension and meeting the transitivity requirement. (2) Although there was no uniform grading, we carefully examined the baseline characteristics of all included studies, which included children with mild-to-moderate persistent asthma. Therefore, there was good consistency in the characteristics of the study population in terms of disease severity. (3) There were differences in specific drug regimens among the studies, but these differences reflected the conventional range of practice for stepwise treatment of childhood asthma in the real world, and the core treatment principles were consistent. Therefore, we believe that good consistency was maintained among the studies on key patient characteristics, supporting the transmissibility hypothesis. The findings of this study emphasize the effect of *probiotics* as an add-on therapy to conventional treatment, and the background differences of conventional treatment should be taken into account when interpreting, with conclusions made cautiously.

Based on the SUCRA ranking analysis, conventional treatment combined with *Bifidobacterium*–*Lactobacillus* triplex live bacteria has the highest probability of being the optimal intervention in terms of increasing FEV_1_% and reducing recurrence rate. Studies (48, 49) suggest that it may promote asthma recovery by suppressing inflammatory markers such as serum chemokine-like factor-1 and nerve growth factor, and by enhancing dendritic cell (DC) and T-cell activity to regulate immune function. Conventional treatment combined with *Bifidobacterium adolescentis* has the highest probability of being the optimal intervention in increasing FEV_1_. This may be related to its upregulation of CD86 expression to promote DC maturation and stimulate DC secretion of IL-12 and IFN-γ, thereby altering Th2 dominant differentiation and correcting Th1/Th2 imbalance (50). Conventional treatment combined with *Lactobacillus* tablets has the highest probability of being the optimal intervention in increasing PEF. Animal studies (51) support lactic acid bacteria in improving asthma responses by inducing IL-10 production, downregulating Th1/Th2 responses, and inhibiting eosinophilic inflammation. Conventional treatment combined with *Bacillus subtilis* diplex live bacteria has the highest probability of being the optimal intervention in terms of improving the clinical total effective rate. Conventional treatment combined with *Bifidobacterium* quadruplex live bacteria has the highest probability of being the optimal intervention in reducing IL-4 and IL-33 levels. Conventional treatment combined with *Bifidobacterium* triplex live bacteria has the highest probability of being the optimal intervention in improving the C-ACT score. It is notable that, with the exception of a few outcome measures such as IgA and FEV_1_, most SUCRA ranking differences were not statistically significant (95% CI overlap rate >75%). Therefore, when making clinical decisions, the uncertainty reflected by the probability ranking results and the range of confidence intervals should be taken into account.

### 4.1 Comparison with other network meta-analyses

Over the past decade or so, studies on *probiotics* for childhood asthma have mainly focused on preventive effects and the exploration (52–54) of mechanisms of action, and there is no meta-analysis similar to this one. Western studies have focused more on early life intervention to prevent asthma development (developmental origins hypothesis), while the included studies mainly addressed clinical management of children with diagnosed asthma (primarily Chinese children). Due to the scarcity of eligible Western studies, the results cannot be directly extrapolated to Western populations at present.

### 4.2 Strengths and limitations of this study

#### 4.2.1 Strengths

This study innovatively compares the regimens and efficacy of different types of *probiotics* in the treatment of childhood asthma, providing evidence-based medical support for standardizing the application of *probiotics* in the treatment of this disease. Excluding children under 5 years ensured diagnostic accuracy and internal validity, though this limits applicability to early childhood when probiotic interventions may have greater immunomodulatory potential. To ensure homogeneity and internal effectiveness of the included studies, we strictly limited the age range to 5–18 years. Asthma phenotypes, diagnostic criteria, and outcome measurement methods were relatively more consistent in children within this age group. Including groups with a wide age range, such as infants and adolescents, would introduce uncontrollable sources of heterogeneity, making the interpretation of the combined effect size ambiguous or even misleading. The 5–18 age group represents an important and large subgroup in asthma management, and its treatment effect assessment is equally instructive for clinical practice. Our study fills the evidence gap for this specific age group. The reliability of the study method was demonstrated through meta-regression and subgroup analyses of interventions, strain counts, etc.

#### 4.2.2 Limitations

Although this study included studies with a sample size of more than 30 cases, which was somewhat credible, there were still limitations. (1) The predominance of Chinese studies (33/34) limits generalizability to Western populations. Our comprehensive search strategy included major English databases (PubMed, Web of Science); the low yield of English studies reflects the current evidence landscape rather than selective searching. (2) Future systematic reviews should prioritize inclusion of international studies to enable robust cross-cultural comparisons. (3) Differences in dosing time, dose–course, follow-up time, and evaluation indicators among different studies led to high heterogeneity in the meta-analysis. The total sample size of the included studies was small. (4) Excluding children <5 years ensures diagnostic accuracy but limits applicability to early childhood, when probiotic interventions may have greater immunomodulatory potential. There is an urgent need for well-designed RCTs in the future to use age-appropriate validation indicators to evaluate the efficacy of *probiotics* for asthma (or recurrent wheezing) in infants and young children.

In addition, 12 studies used multi-strain probiotic formulations analyzed as combined interventions, as individual strain contributions could not be separated. Most included studies used compound probiotic preparations where synergistic or antagonistic effects between strains remain unexplored, making it difficult to attribute efficacy to specific strains. Future studies need to strengthen strain-specific design and delve deeper into the mechanisms and clinical effects of individual strains to provide more precise guidance for application**.** Four interventions were supported by single studies only, limiting confidence in their rankings. Interventions supported by ≤2 studies require confirmatory trials before clinical recommendations.

The observed differences in SUCRA rankings may reflect the combined effects of strain properties, dosing, duration, and patient factors rather than strain-specific mechanisms alone. Direct mechanistic comparisons require specifically designed trials with biomarker assessments.

Some of the randomized controlled studies published domestically failed to describe in detail the random allocation method, allocation concealment, and blinding or had problems with the research methods themselves, which to some extent affected the methodological quality of the studies and posed a risk of bias in the results. Therefore, higher-quality, standardized design, large-sample, RCTs are still needed to evaluate the actual efficacy of *probiotics* to provide better evidence for *probiotics* in the treatment of childhood asthma.

### 4.3 Prospects for the future

Traditional Chinese medicine has unique advantages in treating childhood asthma (such as personalized herbal medicine, simple and well-tolerated external treatment methods), and related research is increasing. However, no RCTs of *probiotics* combined with traditional Chinese medicine or external treatment of traditional Chinese medicine for childhood asthma were found in this search, and the combined effect remains unknown and awaits exploration. This study confirmed the effectiveness of *probiotics* as an adjunctive treatment for childhood asthma and provided a direction for strain selection. Future research should focus on the following areas. (1) Fill the evidence gap: There is an urgent need to conduct high-quality RCTs to evaluate the efficacy of *probiotics* in infantile asthma/recurrent wheezing using age-appropriate indicators. (2) In-depth mechanism and precision: Dose-standardization studies should be conducted to clarify strain-specific dose-effect relationships. (3) Exploring combination options: further research should investigate *probiotics* and traditional Chinese medicine (internal/external) combination therapy to provide a more comprehensive and effective treatment strategy for childhood asthma by advancing research to a deeper and more precise level.

## Data Availability

The original contributions presented in the study are included in the article/Supplementary Material, further inquiries can be directed to the corresponding authors.
